# Radioiodinated PARP1 tracers for glioblastoma imaging

**DOI:** 10.1186/s13550-015-0123-1

**Published:** 2015-09-04

**Authors:** Beatriz Salinas, Christopher P. Irwin, Susanne Kossatz, Alexander Bolaender, Gabriela Chiosis, Nagavarakishore Pillarsetty, Wolfgang A. Weber, Thomas Reiner

**Affiliations:** Department of Radiology, Memorial Sloan Kettering Cancer Center, 1275 York Avenue, New York, NY 10065 USA; Program in Chemical Biology, Memorial Sloan Kettering Cancer Center, New York, NY 10065 USA; Weill Cornell Medical College, New York, NY 10065 USA

**Keywords:** PARP1, Glioblastoma, ^131^I, ^124^I, PET, SPECT, U87 MG, U251 MG

## Abstract

**Background:**

Although the understanding of the genetic and molecular basis of cancer has advanced significantly over the past several decades, imaging and treatment options for glioblastoma patients have been more limited (N Engl J Med 359:492-507, 2008). This is in part due to difficulties in diagnosing this disease early, combined with its diffuse, infiltrative growth. This study was aimed at the development of a novel diagnostic tool for glioblastoma through the synthesis of a small molecule based on radioiodinated poly(ADP-ribose)polymerase 1 (PARP1) targeted tracers. This PARP1 is a biomarker that is overexpressed in glioblastoma tissue, but has only low expression levels in the healthy brain (Neoplasia 16:432-40, 2014).

**Methods:**

A library of PARP1 inhibitors (iodo-PARPis) was synthesized. Based on their pharmacokinetic properties and nuclear PARP1 binding, the most successful inhibitor was radiolabeled with ^131^I and ^124^I. Biodistribution as well as imaging experiments were performed in orthotopic and subcutaneous mouse models of glioblastoma.

**Results:**

One member of our iodo-poly(ADP-ribose)polymerase 1 (PARP1) inhibitor library, I2-PARPi, shows promising biophysical properties for in vivo application. All synthesized tracers have IC_50_ values in the nanomolar range (9 ± 2–107 ± 4 nM) and were able to inhibit the uptake of a fluorescent PARP1 inhibitor analog (PARPi-FL). I2-PARPi was able to reduce the uptake of PARPi-FL by 78 ± 4 % in vivo. In mouse models of glioblastoma, we show that the radioiodinated inhibitor analog has high uptake in tumor tissue (U251 MG xenograft, tumor, 0.43 ± 0.06 %ID/g; brain, 0.01 ± 0.00 %ID/g; muscle, 0.03 ± 0.01 %ID/g; liver, 2.35 ± 0.57 %ID/g; thyroid, 0.24 ± 0.06 %ID/g). PET and SPECT imaging performed in orthotopic glioblastoma models with [^124^I]- and [^131^I]-I2-PARPi showed selective accumulation in the tumor tissue. These results were also verified using autoradiography of tumor sections, which displayed focal selective uptake of the tracer in the tumor regions as confirmed by histology. The uptake could be blocked through pre-injection of excess unlabeled PARP1 inhibitor (Olaparib).

**Conclusions:**

We have successfully synthesized and radioiodinated the PARP1 selective tracer I2-PARPi. The novel tracer shows selective binding to tumor tissue, both in vitro and in models of glioblastoma, and has the potential to serve as a selective PET imaging agent for brain tumors.

**Electronic supplementary material:**

The online version of this article (doi:10.1186/s13550-015-0123-1) contains supplementary material, which is available to authorized users.

## Background

State-of-the-art cancer care is stricken by our inability to efficiently treat, inhibit, and ultimately cure tumor growth for the vast majority of primary cancers. And while for most types of cancer, the diagnosis and detection of lesions has seen significant improvements over the past decades [3], some are often detected at late stages, when therapeutic options are limited. Therefore, early detection remains an unmet clinical need. This is also the case for glioblastoma, the most common cancer in the adult brain. Malignant gliomas are diffusely infiltrative, which limits the use of current diagnostic tools, typically MRI, to detect small pockets of tumor cells, which have invaded the healthy brain [1, 4–7]. Therefore, better and more specific glioblastoma imaging agents with higher signal/noise ratios are needed, which would enable more accurate detection and delineation of infiltrative tumor regions, ultimately leading to better resection and surgical outcomes [7, 8]. Our previous work has shown that small molecular PARP1 inhibitors, like the fluorescent PARPi-FL, accumulate in the nucleus of glioblastoma cells at high concentrations and with high specificity [2]. PARP1 is a near-ideal imaging target for glioblastoma. It holds a unique role in maintaining the integrity of the genome, and the enzyme is one of the key players responsible for DNA repair [7, 9–11]. As a cellular response to DNA damage, PARP1 is activated [12–15]. Due to rapid proliferation, genetic instability, and metabolic stress, DNA damage is more likely to happen in cancer cells than healthy tissue [11]. As a result, PARP1 is highly overexpressed in various forms of cancer [16–21]. This is particularly apparent in mouse models of glioblastoma, where tumor tissue is overexpressing PARP1, whereas healthy brain tissue has very low levels of the enzyme. This drastic difference in expression levels was also observed in human tissue [19] and led us to believe that a non-invasive imaging agent for PARP1 could be used to detect glioblastoma with high signal/noise ratios.

The goal of this project was therefore to develop a non-invasive PET-based imaging platform for PARP1. Based on our successful optical PARP1 imaging agents, we chose a 2H-phthalazin-1-one scaffold [22] previously used by us for targeting glioblastoma cells [23]. We describe the synthesis of a targeted PARP1 tracer, which was labeled with the radioisotopes ^131^I and ^124^I (for SPECT and PET imaging, respectively). Specifically, we show the design of small molecular targeted agents and their validation in biochemical assays, both in cells and in vivo. We further use one tracer for ^131^I/^124^I SPECT and PET imaging of PARP1 in subcutaneous and orthotopic mouse models of cancer, thereby illustrating that the expression of PARP1 is highly upregulated and that our labeled tracer accumulates inside the nuclei of glioblastoma cells, where PARP1 is expressed.

## Methods

Unless otherwise noted, all reagents were purchased from Sigma-Aldrich (St. Louis, MO) and used without further purification. *N*-succinimidyl-4-(tributylstannyl) benzoate was purchased from Synthonix (Cambridge, UK), 3-(3-iodophenyl)propionic acid and 3-(4-iodophenyl)propionic acid from Matrix Scientific (Columbia, SC), and 4-iodophenyl acetic acid from Alfa Aesar (Cambridge, UK). Olaparib (AZD2281) was purchased from LC Laboratories (Woburn, MA). 4-(4-Fluoro-3-(piperazine-1-carbonyl)benzyl)phthalazin-1(2H)-one was synthesized as described previously [22]. ^1^H-nuclear MR (NMR) spectra were recorded at room temperature on a Bruker Avance 500 instrument operating at the frequency of 500 MHz (Billerica, MA) and internally referenced to the residual solvent peaks, CDCl_3_ (7.26 ppm) or dimethyl sulfoxide (DMSO)-d_6_ (2.49 ppm). Mass spectroscopy data was recorded on a Waters Acquity Ultra Performance LC (Milford, MA). High-resolution mass data was recorded on a Waters LCT Premier XE mass spectrometer. High-performance liquid chromatography (HPLC) and radio-HPLC was performed on a Shimadzu HPLC system equipped with 2LC-10AT pumps and an SPD-M10AVP photodiode array detector (Columbia, MD). Radio-HPLC was performed using an identical Shimadzu system, additionally equipped with a Lablogic Scan-RAM Radio-TLC/HPLC detector (Brandon, FL). Analytic runs were performed on a C18 Waters Atlantis T3 column (6 × 250 mm, 5 mm). The solvent system included water (solvent A) and acetonitrile (AcN) (solvent B) for the purification and quality control of the radiotracers with a gradient of 5–95 % B between 0 and 15 min and 100 % B between 15 and 25 min. For the purification of non-radioactive precursors, water (0.1 % trifluoroacetic acid, solvent A) and acetonitrile (AcN) (0.1 % trifluoroacetic acid, solvent B) were used, all with a flow rate of 1 mL/min and a gradient of 5–95 % B between 0 and 15 min, 95 % B between 15 and 17 min, and 95–5 % B between 17 and 18 min.

### Synthesis of PARP1 inhibitors

#### 4-(4-fluoro-3-(4-(3-iodobenzoyl)piperazine-1-carbonyl)benzyl)phthalazin-1(2H)-one (I1-PARPi)

To a solution of 4-(4-Fluoro-3-(piperazine-1-carbonyl)benzyl)phthalazin-1(2H)-one (10 mg, 0.0275 mmol), triethylamine (40 μL, 0.3 mmol) and HBTU (16 mg, 0.0413 mmol) in dimethyl formamide (DMF, 500 μL) were added to 3-iodobenzoic acid (6 mg, 0.0275 mmol). The mixture was stirred at room temperature for 20 h. The crude product was then purified by preparative HPLC and dried under vacuum, yielding a white solid (6.9 mg, 48 % yield). ^1^H-NMR (CDCl_3_) *δ* = 10.00 (s, 1H), 8.40–8.38 (m, 1H), 7.71–7.69 (m, 4H), 7.64–7.63 (m, 1H), 7.30–7.26 (m, 3H), 7.09 (m, 1H), 7.04–6.87 (m, 1H), 4.21 (s, 2H), 3.71–3.29 (m, 8H). LC-ESI-MS (+) m/z = 597.1 [M+H^+^]^+^. HRMS-ESI [M-H^+^]^−^ m/z calculated for [C_27_H_22_FIN_4_O_3_]^−^ 595.0642, found 595.0660.

#### 4-(4-fluoro-3-(4-(4-iodobenzoyl)piperazine-1-carbonyl)benzyl)phthalazin-1(2H)-one (I2-PARPi)

A solution of 4-(4-fluoro-3-(piperazine-1-carbonyl)benzyl)phthalazin-1(2H)-one (10 mg, 0.0275 mmol), HBTU (16 mg, 0.0413 mmol), triethylamine (40 μL, 0.3 mmol), and 4-iodobenzoic acid (6 mg, 0.0245 mmol) in DMF (500 μL) was stirred overnight at room temperature. The crude product was purified by preparative HPLC and dried under vacuum, yielding a white solid (8.8 mg, 61 % yield). ^1^H-NMR (CDCl_3_) *δ* = 10.48 (s, 1H), 8.40–8.39 (m, 1H), 7.74–7.66 (m, 5H), 7.27–7.26 (d, 2H), 7.09–7.07 (d, 2H), 4.22 (s, 2H), 3.73–3.14 (m, 8H). LC-ESI-MS (+) m/z = 597.1 [M+H^+^]^+^. HRMS-ESI [M-H^+^]^−^ m/z calculated for [C_27_H_22_FIN_4_O_3_]^−^ 595.0642, found 595.0640.

#### 4-(4-fluoro-3-(4-(2-(3-iodophenyl)acetyl)piperazine-1-carbonyl)benzyl)phthalazin-1(2H)-one (I3-PARPi)

A solution of 3-iodophenyl acetic acid (6.5 mg, 0.048 mmol), 1-ethyl-3-(3-dimethylaminopropyl) carbodiimide (EDC) (10.5 mg, 0.055 mmol), *N*-hydroxy succinimide (NHS), and 600 μL DMF was stirred for 30 min at room temperature. Then, 4-(4-fluoro-3-(piperazine-1-carbonyl)benzyl)phthalazin-1(2H)-one (10 mg, 0.0275 mmol) was added to the solution, and the mixture was stirred at room temperature overnight. The reaction was washed with 500 μL of H_2_O and extracted with 500 μL dichloromethane (DCM). The resulting organic solution was purified on silica gel, using a gradient elution from neat DCM to DCM/hexane 5:1 to obtain the desired product as a white solid (3 mg, 20 % yield). ^1^H-NMR (CDCl_3_) *δ* = 9,82 (s, 1H), 8.40–8.38 (m, 1H), 7.71–7.69 (m, 2H), 7.55–7.53 (m, 1H), 7.51–7.50 (m, 2H), 7.25–7.24 (m, 2H), 7.09–6.90 (m, 3H), 4.20 (s, 2H), 3.64–3.31 (m, 8H), 2.84 (s, 2H). LC-ESI-MS (+) m/z = 633.1 [M+Na^+^]^+^. HRMS-ESI [M+H^+^]^+^ m/z calculated for [C_28_H_24_FIN_4_O_3_]^+^ 611.0955, found 611.0948.

#### 4-(4-fluoro-3-(4-(2-(4-iodophenyl)acetyl)piperazine-1-carbonyl)benzyl)phthalazin-1(2H)-one (I4-PARPi)

A solution of 4-iodophenyl acetic acid (6.5 mg, 0.048 mmol), EDC (10.5 mg, 0.055 mmol), NHS, and 600 μL DMF was stirred for 30 min at room temperature. After this time, the 4-(4-fluoro-3-(piperazine-1-carbonyl)benzyl)phthalazin-1(2H)-one (10 mg, 0.0275 mmol) was added to the solution and the mixture was stirred at room temperature overnight. H_2_O (500 μL) was added, the mixture was extracted with DCM (2 × 500 μL), and the combined extracts were dried under vacuum. The crude mixture was purified by silica column chromatography (100 % DCM), and the product obtained was a white solid (8.8 mg, 61 %). ^1^H-NMR (CDCl_3_) *δ* = 9.82 (s, 1H), 8.40–8.38 (m, 1H), 7.83–7.81 (d, 1H), 7.77–7.75 (d, 1H), 7.70–7.69 (m, 2H), 7.63–7.56 (m, 3H), 7.00–6.89 (m, 3H), 4.20 (s, 2H), 3.63–3.11 (m, 8H), 2.84 (s, 2H). LC-ESI-MS (+) m/z = 632.9 [M+Na^+^]^+^. HRMS-ESI [M+H^+^]^+^ m/z calculated for [C_28_H_24_FIN_4_O_3_]^+^ 611.0955, found 611.0971.

#### 4-(4-fluoro-3-(4-(3-(3-iodophenyl)propanoyl)piperazine-1-carbonyl)benzyl)phthalazin-1(2H)-one (I5-PARPi)

A solution of 4-(4-fluoro-3-(piperazine-1-carbonyl)benzyl)phthalazin-1(2H)-one (10 mg, 0.0275 mmol), HBTU (16 mg, 0.0413 mmol), triethylamine (40 μL, 0.3 mmol), and 3-(3-iodophenyl)propionic acid (7.6 mg, 0.0275 mmol) in 400 μL of AcN was stirred overnight at room temperature. The crude product was then purified by preparative HPLC and the isolated product dried at vacuum to obtain a white solid (5.1 mg, 38 %). ^1^H-NMR (CDCl_3_) *δ* = 10.33 (s, 1H), 8.41–8.39 (d, 1H), 7.71–7.63 (m, 3H), 7.51–7.45 (m, 2H), 7.27–7.25 (m, 2H), 7.12–6.92 (m, 3H), 4.22 (s, 2H), 3.65–3.12 (m, 8H), 2.88–2.83 (m, 2H), 2.59–2.48 (m, 2H). LC-ESI-MS (+) m/z = 647.1 [M+Na^+^]^+^. HRMS-ESI [M+H^+^]^+^ m/z calculated for [C_29_H_26_FIN_4_O_3_]^+^ 625.1112, found 625.1111.

#### 4-(4-fluoro-3-(4-(3-(4-iodophenyl)propanoyl)piperazine-1-carbonyl)benzyl) phthalazin-1(2H)-one (I6-PARPi)

4-(4-Fluoro-3-(piperazine-1-carbonyl)benzyl)phthalazin-1(2H)-one (10 mg, 0.0275 mmol) was mixed with HBTU (16 mg, 0.0413 mmol), triethylamine (40 μL, 0.3 mmol), and 4-iodo-3-phenyl propionic acid (7.6 mg, 0.0275 mmol) in 400 μL of AcN, and the solution was stirred overnight at room temperature. The crude product was then purified by preparative HPLC and the isolated product dried at vacuum to obtain a white solid (7.5 mg, 45 %). ^1^H-NMR (CDCl_3_) *δ* = 9.71 (s, 1H), 8.40–8.38 (d, 1H), 7.70–7.69 (m, 2H), 7.64–7.63 (m, 1H), 7.55–7.52 (m, 2H), 7.27–7.25 (m, 2H), 7.00–6.97 (m, 1H), 6.91–6.87 (m, 2H), 4.20 (s, 2H), 3.64–3.11 (m, 8H), 2.87–2.85 (m, 2H), 2.63–2.47 (m, 2H). LC-ESI-MS (+) m/z = 647.1 [M+Na^+^]^+^. HRMS-ESI [M+Na^+^]^+^ m/z calculated for [C_29_H_26_FIN_4_O_3_Na]^+^ 647.0931, found 647.0941.

### Radiochemistry

[^131^I]-NaI was purchased at Nordion (Ottawa, ON, Canada) in NaOH solution (0.1 M) with a concentration of 0.99–2.5 mCi/μL. [^124^I]-NaI was produced at Memorial Sloan-Kettering Cancer Center (New York, NY) in NaOH solution (0.5 M) with a concentration of 0.20–0.40 mCi/μL.

#### Synthesis of [^131^I]-NHS-benzoate

Precursor *N*-succinimidyl-4-(tributylstannyl) benzoate (30 μg, 5.9 nmol) was dissolved in 30 μL of AcN, and the solution was added to a solution of methanol (40 μL), chloramine T (6 μg, 0.03 nmol) in acetic acid (2 μL), and [^131^I]-NaI in NaOH 0.1 M (1–2.5 mCi). After 5 min at room temperature, the reaction was purified by HLPC on a C18 Waters Atlantis T3 column (6 × 250 mm, 5 mm), using water (solvent A) and AcN (solvent B) as mobile phase, with an elution gradient from 5 to 100 % for solvent B over 15 min and then 100 % of solvent B from 15 to 25 min. The retention time of [^131^I]-NHS benzoate was 14.3 min, and its identity was established by co-elution with the reference cold compound. The radiochemical yield was 67 ± 6 % (*n* = 12), and the radiochemical purity was >98 %. The collected fraction containing [^131^I]-NHS benzoate was concentrated to dryness under vacuum.

The same procedure was followed for the synthesis of [^124^I]-NHS benzoate. In this case, the radiochemical yield was 32 ± 5 % (*n* = 5) and the purity was >95 %.

#### Synthesis of [^131^I]-I2-PARPi

The dried radiolabeled [^131^I]-NHS-benzoate precursor was dissolved in 200 μL of AcN, and an excess of HBTU (1 mg, 2.6 nmol) and 4-(4-fluoro-3-(piperazine-1-carbonyl)benzyl)phthalazin-1(2H)-one (1 mg, 2.7 nmol) was added and allowed to react for 3 h at 32 °C. The final product was purified by HPLC, using water (solvent A) and AcN (solvent B) as solvents with a gradient elution from 5 to 100 % of solvent B over 15 min and then 100 % of B from 15 to 25 min. The retention time of [^131^I]-I2-PARPi was 13.1 min, and its identity was established by co-elution with the reference cold compound. The radiochemical yield was 72 ± 8 % (*n* = 12) and the radiochemical purity >95 %. The collected fraction containing [^131^I]-I2-PARPi was concentrated to dryness under reduced pressure.

The same procedure was followed for the synthesis of [^124^I]-I2-PARPi. In this case, the radiochemical yield was 68 ± 5 % (*n* = 5) and the purity >95 %.

### Cell culture

The human glioblastoma cell lines U251 MG and U87 MG were generously provided by the Laboratory of Dr. Ronald Blasberg (MSKCC, New York, NY). All cell lines were grown in Eagle’s minimal essential medium (MEM) containing 10 % (*v*/*v*) heat-inactivated fetal bovine serum, 100 IU penicillin, and 100 μg/mL streptomycin. Cells were cultured at 37 °C in a humidified 5 % CO_2_ atmosphere. All media was purchased from the media preparation facility at MSKCC (New York, NY).

### Mouse models

Six 10-week-old female athymic nude CrTac:NCr-Fo mice from Taconic Laboratories (Hudson, NY) were used for all mouse experiments. During subcutaneous injections, mice were anesthetized using 2 % isoflurane gas in 2 L/min medical air. During orthotopic injections, mice were anesthetized using a 150 mg/kg ketamine and 15 mg/kg xylazine cocktail (10 μL/g). Before all intravenous injections, mice were gently warmed with a heat lamp and placed in a restrainer and tails were sterilized with alcohol pads. The lateral tail vein was used for all intravenous injections. All mouse experiments were done in accordance with protocols approved by the Institutional Animal Care and Use Committee of MSKCC and followed National Institutes of Health (NIH) guidelines for animal welfare.

### PARP-1 IC_50_ determination

A commercially available colorimetric assay (Trevigen, Gaithersburg, MD) was used to measure PARP-1 activity in vitro in the presence of varying concentrations of the different iodo-PARPis. Specifically, dilutions of iodo-PARPi (final concentrations ranging from 3.3 μM to 0.1 nM) were incubated with 0.5 U of PARP1 high specific activity (HSA) enzyme for 10 min in histone-coated 96-well plates. All experiments were carried out in triplicate. Positive control samples did not contain inhibitor, and negative control samples did not contain PARP1. All reaction mixtures were adjusted to a final volume of 50 μl, and a final concentration of 1 % DMSO in assay buffer. The remainder of the assay was performed according to the manufacturer’s instructions. PARP1 activity was measured by absorbance at 450 nm in each well using a SpectraMax M5 spectrophotometer with SoftMax Pro software (Molecular Devices, Sunnyvale, CA).

### Hydrophobicity index determination

Chemical hydrophobicity indices (CHIs) were measured using procedures developed previously [24]. Briefly, reverse phase HPLC was used to measure the retention times of a set of standards with known CHI. A standard curve was then created to calculate the CHIs of all iodo-PARPi based on the HPLC retention time. Log *P* values were derived from CHI values following the equation: Log *P* = 0.0566 ± CHI − 1.107.

### Plasma protein fraction

The plasma protein fraction was determined using the Rapid Equilibrium Dialysis Device System (Life Technologies, Grand Island, NY) according to the manufacturer’s protocol. Membrane dialysis was performed with 10 μM of compound in mouse serum (500 μL) on one side of the membrane and PBS (750 μL) on the other side. The system was sealed with parafilm and incubated for 4 h at 37 °C on an orbital shaker set to 250 rpm. Thereafter, 400 μL of solution was taken from both sides, and samples were treated twice with an equal amount of AcN and vortexed to remove protein before HPLC analysis. After injection (100 μL), the I-PARPi peaks from each sample were then integrated and the protein bound fraction was determined. The data was analyzed using Prism 6.0c.

### Immunohistochemistry

#### PARP1 expression in tissues

PARP1 antigen detection in glioblastoma xenografts and mouse brain was performed at MSKCC’s Molecular Cytology Core Facility using the Discovery XT processor (Ventana Medical Systems, Tucson, AZ) and detected using immunofluorescence (IF) staining. Paraffin-embedded formalin-fixed 3 μm sections were deparaffinized with EZPrep buffer, antigen retrieval was performed with CC1 buffer (both Ventana Medical Systems), and sections were blocked for 30 min with Background Buster solution (Innovex, Richmond, CA). Anti-PARP1 rabbit polyclonal antibody (sc-7150, 0.2 μg/mL; Santa Cruz Biotechnology, Santa, Cruz, CA) was incubated for 5 h, followed by 1 h incubation with biotinylated goat anti-rabbit IgG (Vector labs, PK6106) at a 1:200 dilution. Detection was performed with Streptavidin-HRP D (from DABMap Kit, Ventana Medical Systems), followed by incubation with Tyramide Alexa Fluor 594 (T20935; Invitrogen, Carlsbad, CA) prepared according to the manufacturer’s instructions. Sections were counterstained with 4′,6-diamidino-2-phenylindole (DAPI) and coverslipped with Mowiol® mounting medium (Sigma-Aldrich, St. Louis, MO). H&E staining was performed on adjacent sections for morphological evaluation of tissue characteristics.

### Quantification of PARP1 expression

Protein expression was quantified on digitalized PARP1-stained sections using at least ten fields of view per section. Thresholding of the blue (nuclei stained with DAPI) and red fluorescent area (nuclei stained with PARP1) was performed using MetaMorph® Software (Molecular Devices, Sunnyvale, CA). PARP1 intensity was determined by measuring the red fluorescence intensity in the area of all nuclei, and the % PARP1 positive nuclear area was calculated by dividing the PARP1 positive area by the DAPI positive area in each field of view.

### In vitro blocking study

U87 MG cells were seeded into a 96-well plate in a concentration of 1 × 10^4^ cells per well. After 24 h, the cells were incubated with either the fluorescent PARP1 inhibitor PARPi-FL (250 nM) alone or with one of the iodo-PARPi inhibitors at a 100-fold higher concentration (25 μM) for 20 min. Additionally, as a positive control, the PARP1 inhibitor Olaparib was used. All incubation solutions also included Hoechst 33342 nuclear stain (Sigma-Aldrich, St. Louis, MO). The cells were washed twice with media and once with PBS for 5 min each and imaged on an LSM 5Live confocal microscope (Zeiss, Oberkochen, Germany). All wells were imaged with the DAPI filter for the Hoechst staining and the FITC filter for the PARPi-FL staining. The DAPI and FITC channels were co-registered, and the green fluorescence in the location of the Hoechst staining was quantified for each image. The percent reduction in PARPi-FL uptake was calculated based on the level of fluorescence intensity seen in each image and normalized to the cells receiving no iodo-PARPi inhibitor. Experiments were performed in triplicate.

### Blood half-life

Blood half-life was determined by measuring the activity in serial blood samplings. Specifically, healthy female nude mice (8–10 weeks old, 20–25 g in weight, *n* = 3) were injected via the tail vein with 50 μCi [^131^I]-I2-PARPi in 200 μL of solution PBS/PEG_300_ (10:1). The blood was sampled from the saphenous vein at 5, 15, 30, 60, 120, and 240 min post injection. The blood was weighed and radioactivity was measured on a Wizard 2470 Automatic Gamma Counter (Perkin Elmer, Waltham, MA). Measurements in counts per minute were calculated as the mean %ID/g. The blood half-life was calculated using Prism 6.0c (GraphPad Software, La Jolla, CA).

### In vivo blocking study

To verify the specificity of tumor uptake of I2-PARPi in vivo, the level of blocking of the fluorescent PARP1 inhibitor on a macroscopic and microscopic scale was determined. Nude mice bearing subcutaneous U87 MG tumors were injected with either the fluorescent PARP1 inhibitor PARPi-FL alone (2.5 mg/kg, 200 μL of 19.5 % 1:1 DMAC:Kolliphor, 3.5 % DMSO, 77 % PBS), PARPi-FL 30 min after a pre-injection of a 50-fold excess of I2-PARPi (125 mg/kg, 100 μL of 10 % PEG_300_, 90 % PBS), or injected with saline alone. One hour post injection, the mice were sacrificed and the tumors were resected and imaged with the IVIS spectrum fluorescence imaging system (PerkinElmer, Waltham, MA) using Living Image 4.4 software. The tumors were also imaged microscopically with the 5Live fluorescent confocal microscope using the 488 nm laser for PARPi-FL excitation.

### In vitro whole blood stability

The in vitro stability was assessed by incubating 6 μCi [^131^I]-PARPi in mouse blood for 0 to 60 min at 37 °C. At baseline, 15, 30, and 60 min, the samples were immediately placed on ice and mixed 1:1 with a solution of AcN/DMSO (250 μL) and then vigorously vortexed for 30 s to precipitate out serum protein. The sample was centrifuged at 3000 RCF for 3 min at 4 °C, and the supernatant was collected. This procedure was repeated three times, and the combined supernatants were analyzed by HPLC equipped with radioactive detector (Shimadzu, Kyoto, Japan), collecting samples every 30 s. Radioactivity of each fraction was measured on a Wizard 2470 Automatic Gamma Counter (Perkin Elmer, Waltham, MA), and the blood stability was analyzed using Prism 6.0c (GraphPad Software, La Jolla, CA).

### Biodistribution studies

Biodistribution experiments were conducted on female nude mice (8–10 weeks old and 20–25 g in weight, *n* = 21) bearing U87 MG or U251 MG subcutaneous xenografts. The radiolabeled small molecule preparation (30–20 μCi of [^131^I]-I2-PARPi in 200 μL of a solution 90 % PBS 10 % PEG_300_) was administrated via the lateral tail vein. To determine the optimal specific activity to achieve the highest tumor to organ ratio, various specific activities were tested (5, 50, and 250 mCi/μmol) in mice bearing U87 MG tumors. The compound was allowed to circulate for 2 h post injection at which time the mice were sacrificed and organs were harvested (*n* = 3). After determining the optimal specific activity, the optimal time for imaging was determined by testing the drug distribution in nude mice bearing U87 MG tumors at different time points. The drug was allowed to circulate for various times (1, 2, and 6 h), after which the mice were sacrificed (*n* = 3). The radioactive content in the tissue of interest (blood, tumor, muscle, bone, liver, spleen, kidney, heart, lung, pancreas, brain, skin, small intestine, large intestine, stomach, tail, thyroid, and feces) was measured on a Wizard 2470 Automatic Gamma Counter and the tissue-associated activity was calculated as the mean %ID/g.

### Autoradiography

U251 MG glioblastoma cells (5 × 10^4^ in 2 μL of PBS) were orthotopically implanted in athymic nude mice, using a stereotaxic device, and the tumors were allowed to grow for approximately 4 weeks. Once tumors reached the sufficient size, the orthotopic U251 MG tumor-bearing mice were injected intravenously with 500 μCi [^131^I]-PARPi (in 200 μL of a solution PBS 90 % PEG_300_ 10 %, *n* = 2) alone or with a pre-injection of 15 μmol Olaparib (in 100 μL of 7.5 % DMSO, 12.5 % PEG_300_, 80 % PBS) 30 min prior to the injection of [^131^I]-PARPi. Additionally, healthy mice were also injected with 500 μCi [^131^I]-PARPi. After 2 h of circulation time after the [^131^I]-PARPi injection, the mice were sacrificed. Liver, tumor, muscle, and brain tissues were excised and embedded in O.C.T. compound (Sakura Finetek, Torrance, CA) and frozen at −20 °C, and a series of 8 μm frozen sections was cut and mounted on microscope slides. To determine radiotracer distribution, digital autoradiography was performed by placing tissue sections in a film cassette against a phosphor image plate (BASMS-2325; Fujifilm) for 48 h at −20 °C. Phosphor imaging plates were read at a pixel resolution of 25 μm with a Typhoon 7000IP plate reader (GE Healthcare, Piscataway, NJ). After autoradiographic exposure, the same frozen sections were then used for immunohistochemical staining. Areas of brain slides containing tumor tissue were identified using the H&E staining and then overlaid with the autoradiographic data. Intensity of tumor areas and non-tumor areas were then quantified using ImageJ 1.47u.

### In vivo imaging

SPECT/CT was acquired in athymic nude mice (6–10 weeks old). Before administration of the radioiodinated tracer, in terms to block the thyroid, the animals were treated with an intraperitoneal injection of NaI (100 μL, 0.6 mM) 60 min previous to the injection of 450–600 μCi (145–210 mCi/μmol) [^131^I]-I2-PARPi in 200 μL PBS solution (10 % PEG_300_) via the lateral tail vein and then anesthetized with isoflurane mixed with medical air (2 % for induction and maintenance). Animals were placed in prone position, and scans were then performed 90 min after injection for 60 min using a SPECT/CT small animal imaging system (NanoSPECT/CT, Mediso, Boston, MA). SPECT Images were reconstructed using HiSPECT software, and in vivo Scope software was used for CT image reconstruction.

In the case of PET imaging, images were acquired after the injection of 200–250 μCi (110–170 mCi/μmol) [^124^I]-I2-PARPi in 200 μL PBS solution (10 % PEG_300_) via the lateral tail vein under isoflurane anesthesia (2 % for induction and 1.5 % for maintenance). Mice were also treated with an intraperitoneal injection of NaI (100 μL, 0.6 mM), 60 min previous to the administration of the radioiodinated tracer. Animals were immediately placed in prone position under isoflurane anesthesia, and scans were then performed 90 min after injection for 30 min using the Inveon PET/CT imaging system (Siemens, Knoxville, TN). PET and CT Images were reconstructed using Inveon research workplace software.

### Formulation of [^131^/^124^I]-I2-PARPi for in vivo injection

For in vivo applications, the radioactive ^124^I/^131^I-I2-PARPi was injected intravenously, using hypodermic syringes with 200 μL of a solution of PBS 1× and PEG_300_ (9/1 *v*/*v*). We used approximately 450–600 μCi of radiotracer for PET imaging, 200–250 μCi for SPECT imaging, 500 μCi for autoradiography, and 30–20 μCi for biodistributions.

## Results

### Immunohistochemistry

**Fig. 1:**
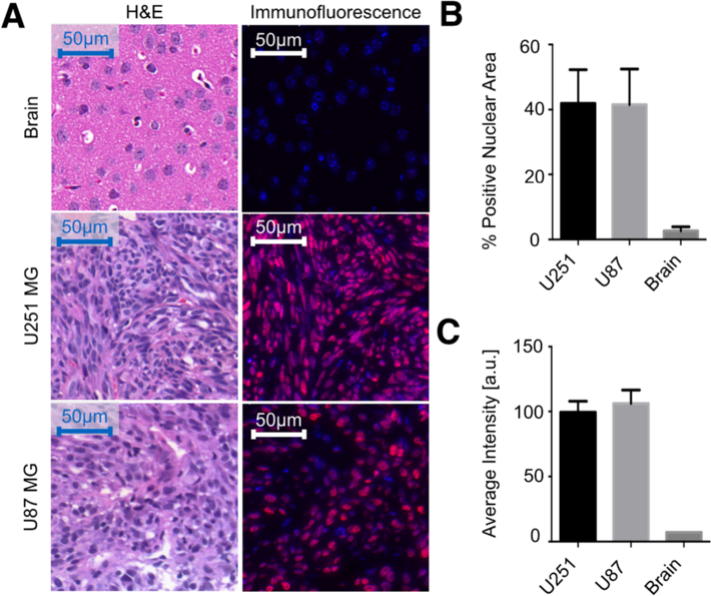
PARP1 immunofluorescence staining of brain, U251 MG xenografts, and U87 MG xenografts. **a** Representative images showing H&E staining (*left*) and PARP1 immunofluorescence staining (*right*) of each tissue type. **b** Percent of nuclei positive for PARP1 for each tissue type. **c** Average intensity of nuclei in each cell type

Immunofluorescence PARP1 antigen detection in histological sections of U251 MG and U87 MG glioblastoma xenografts showed an overexpression of PARP1 compared to healthy brain tissue (Fig. 1a). The PARP1 positive area in glioblastoma slides was increased by a factor of 15.8 ± 8.1 (Fig. 1b). The percent PARP1 positive area in both glioblastoma models was similar, with 42.0 ± 10.4 % and 41.5 ± 11.0 % for U251 MG and U87 MG, respectively (for healthy brain, PARP1 positive area was 2.7 ± 1.3 %). In the same way, glioblastoma tissues present similar values in average intensity (99.5 ± 8.5 AU for U251 MG and 106.2 ± 10.5 AU for U87 MG, Fig. 1c) with values over 14-fold higher than healthy tissue, confirming optimal target/background ratios for in vivo evaluation.

### Synthesis of iodinated PARP1 inhibitors

**Fig. 2:**
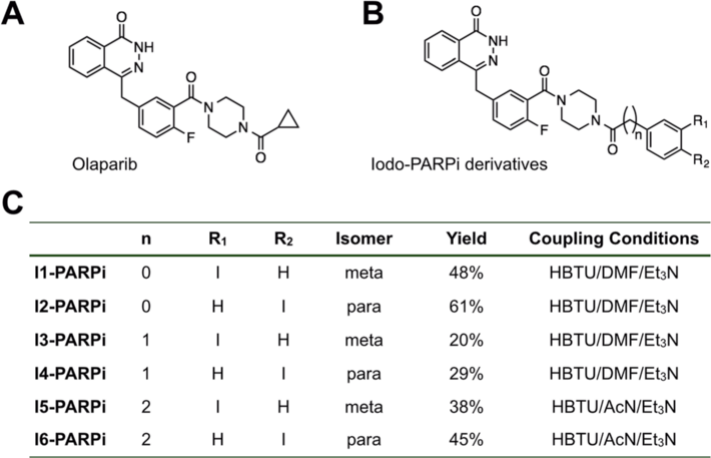
Structure of Olaparib and iodo-PARPi inhibitors. **a** Molecular structure of Olaparib. **b** General structure of I-PARPi derivatives. **c** Molecular structures of I-PARPi derivatives, yields, and synthetic parameters

The molecular structure of all iodinated PARP1 tracers is based on the (2H)-phthalazin-1-one scaffold of the small molecule therapeutic Olaparib (Fig. 2a). The synthesis of a library of test compounds, consisting of six different inhibitors (Fig. 2), was carried out through the coupling of the PARP Inhibitor precursor 4-(4-fluoro-3-(piperazine-1-carbonyl)benzyl)phthalazin-1(2H)-one and different iodinated carboxylic acids. The reaction was performed in the presence of HBTU and triethylamine or EDC and NHS at room temperature, overnight, and crude mixtures were purified by HPLC or silica column, resulting in the isolation of I1-PARPi through I6-PARPi in good yields (Fig. 2b, Additional file 1: Figures S2 and S3).

### Pharmacokinetic properties of non-radioactive compounds

**Fig. 3:**
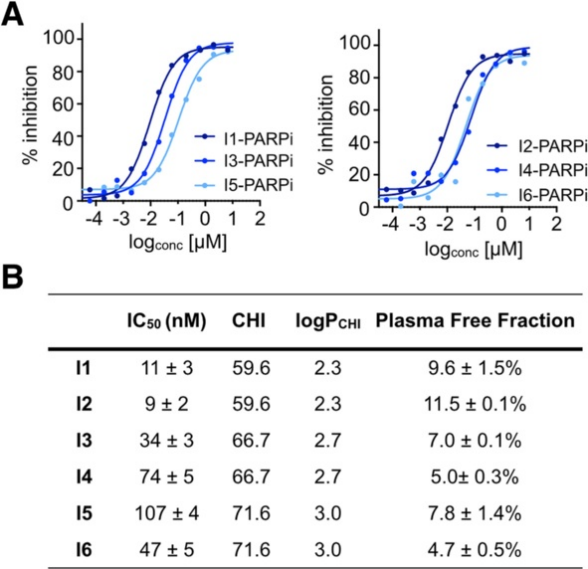
Biochemical parameters and binding affinity of iodo-PARPi derivatives. **a** Percent inhibition of PARP1 using a range of concentrations of the inhibitors I1–I6. **b** Table of IC_50_ values calculated from **a**, along with the values of chromatographic hydrophobicity index (CHI), Log*P*_CHI_, and plasma free fraction

The obtained IC_50_ values of all the small molecules were in the nanomolar range (9 ± 2–107 ± 4 nM). I1-PARPi and I2-PARPi showed the highest affinity (11 ± 3 and 9 ± 2 nM, respectively), with values close to Olaparib (5 nM, [22]). All inhibitors had a CHI between 59.6 and 71.6, which corresponds to Log*P*_CHI_ values between 2.3 and 3.0. These values were lower for Olaparib (CHI = 34.1, Log_CHI_ = 0.8) but adequate for crossing the blood-brain barrier for application in brain diseases [2]. The iodinated molecules showed relatively high plasma protein binding, with the protein plasma free fraction ranging between 4.69 and 11.53 % (Fig. 3).

### In vitro and in vivo competitive optical imaging

**Fig. 4:**
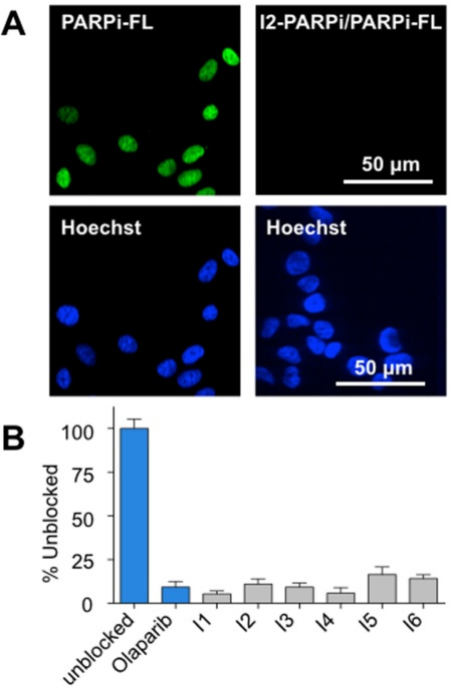
In vitro competitive inhibition of PARPi-FL uptake in U87 MG cells with the different iodo-PARPi derivatives. **a** Representative confocal images of cells stained with PARPi-FL (*green*) and Hoechst 33342 (*blue*) alone (*left*) or stained with PARPi-FL (250 nM) and Hoechst 33342 after addition of iodo-PARPi (25 μM) (*right*). **b** Effectiveness of the iodo-PARPi inhibitors to saturate PARP1, measured in % unblocked of each iodo-PARPi derivative along with the PARP1 inhibitor Olaparib

To demonstrate the specific binding of our inhibitors to PARP1, in vitro competition assays were performed with the small molecules and their fluorescent sister imaging agent, PARPi-FL [25]. PARPi-FL alone led to strong nuclear fluorescence, where the agent was retained by PARP1. The reduction in fluorescent signal in the presence of all inhibitors confirmed their ability to diffuse into the nucleus of the cell and bind PARP1 (Fig. 4a). When PARPi-FL was added to cells that were co-treated with any of the iodo-PARPi agents, a reduction in fluorescence signal between 76 ± 6 and 67 ± 13 % (Fig. 4b) was seen. This was similar to results obtained for Olaparib, where co-treatment resulted in a reduction of PARPi-FL uptake by 73 ± 10 %.

**Fig. 5:**
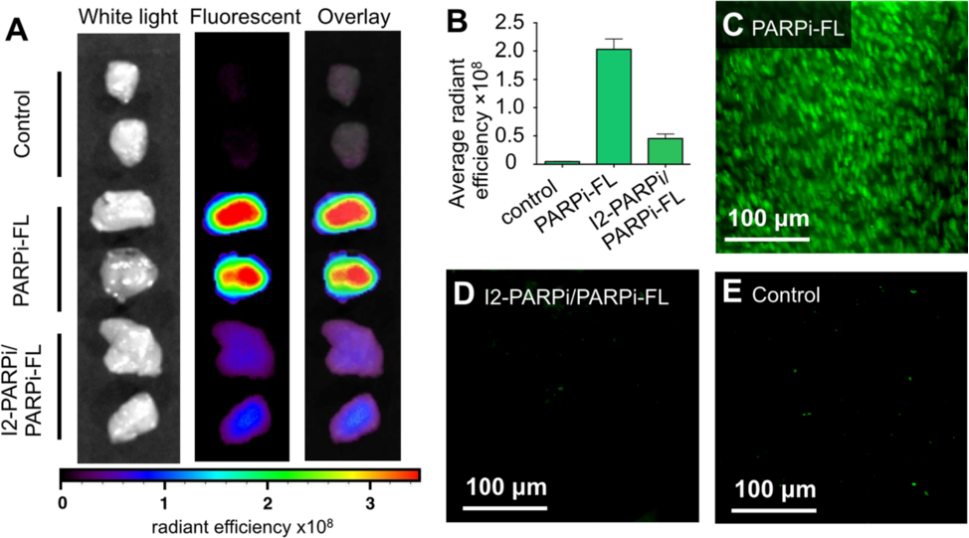
In vivo competitive inhibition of PARPi-FL uptake in U87 MG subcutaneous tumors with I2-PARPi. **a** White light, fluorescent, and overlay images of whole tumor tissues resected from mice treated with PBS (*top*), PARPi-FL alone (2.5 mg/kg, 200 μL of 19.5 % 1:1 DMAC:Kolliphor, 3.5 % DMSO, 77 % PBS) (*middle*), or PARPi-FL (2.5 mg/kg, 200 μL of 19.5 % 1:1 DMAC:Kolliphor, 3.5 % DMSO, 77 % PBS) and I2-PARPi (125 mg/kg, 100 μL of 10 % PEG_300_, 90 % PBS) (*bottom*). **b** Fluorescence intensity of tissues shown in **a**. **c**–**e** Microscopic fluorescence images of tissues shown in **a**

The binding characteristics of non-radioactive I2-PARPi were explored ex vivo by epifluorescence imaging of U87 MG tumor tissue. The tissue was obtained from mice that were injected with I2-PARPi before receiving an injection of PARPi-FL. A second group received PARPi-FL alone, without injection of I2-PARPi (Fig. 5a). Mice receiving both agents showed a 78 ± 4 % lower tumor fluorescence, compared with the mice receiving just PARPi-FL (4.53 × 10^7^ ± 0.81 × 10^7^ and 2.03 × 10^8^ ± 1.84 × 10^7^ average radiant efficiency, respectively). A control group received just PBS, and tumors from these mice did not show significant fluorescence (0.42 × 10^6^ ± 0.07 × 10^6^ average radiant efficiency). Similar results could be seen using confocal microscopy (Fig. 5c–e), where tumors from mice injected with PARPi-FL show clear nuclear uptake in U87 MG tumors (Fig. 5c), whereas there was a significant decrease in uptake for mice which also received I2-PARPi (Fig. 5d), similar to what was observed for the control tumors (Fig. 5e).

### Radiolabeling and stability of [^131^I]-I2-PARPi

**Fig. 6:**
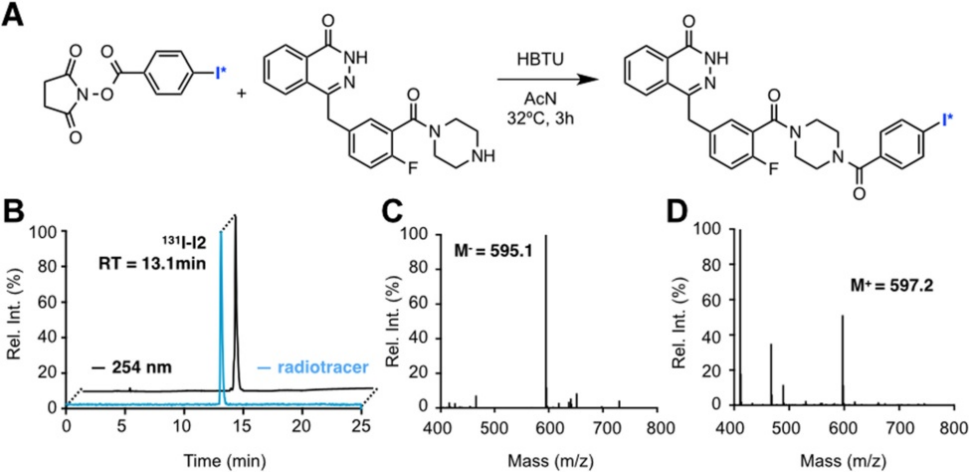
Radiochemical synthesis of I2-PARPi. **a** Coupling reaction with para-iodo NHS-benzoate and PARPi precursor. **b** HPLC chromatogram of [^131^I]-I2-PARPi (radiotrace in *blue*, UV trace in *black*). **c**,**d** Mass spectrometry chromatograms of I2-PARPi

The preparation of the radiotracer [131I]-I2-PARPi was realized in two synthetic steps. First, the precursor *N*-succinimidyl-4-(tributylstannyl) benzoate was labeled in the presence of [131I]-NaI and chloramine T in acetic acid (Additional file 1: Figure S4 A) and isolated by HPLC (Additional file 1: Figure S4 C), with a radiochemical yield of 67 ± 6 % (*n* = 12). The resulting radioactive *N*-succinimidyl-4-(131I-iodo) benzoate was then conjugated to a PARP1 targeting 2H-phthalazin-1-one in the presence of HBTU and AcN at room temperature (Fig. 6a). The crude mixture was purified by HPLC, yielding the pure product with a radiochemical yield of 72 ± 8 % (*n* = 12) and a radiochemical purity >95 %. HPLC chromatograms and mass spectrometry data are shown in Fig. 6b–d. For obtaining [124I]-I2-PARPi, an identical procedure was used, which results in yields of 68 ± 5 % (*n* = 5) and a radiochemical purity >95 %.

In vitro blood stability studies (37 °C) showed only one main peak at 15 min, corresponding to the pure compound [^131^I]-I2-PARPi. We did not observe other major peaks, which would indicate small molecule metabolites, which confirmed the stability of the drug over the course of 120 min (Additional file 1: Figure S5 A–C).

### In vivo pharmacokinetics of [^131^I]-I2-PARPi

**Fig. 7:**
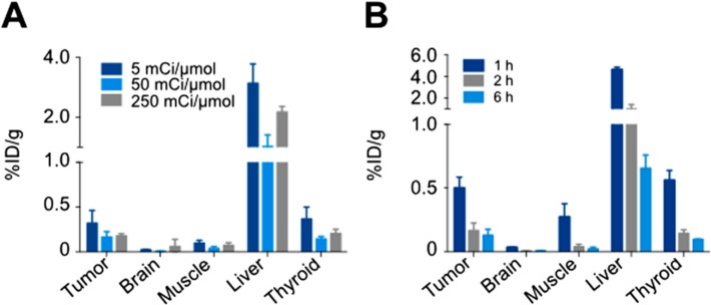
Biodistribution of [^131^I]-I2-PARPi in selected organs. **a** Comparison of the biodistribution of different specific activities of the compound 2 h post injection. **b** Comparison of the biodistribution of the compound at 1, 2, and 4 h post injection. Additional biodistribution data can be found in Additional file 1: Tables S1 and S2

We determined the blood half-life of [^131^I]-I2-PARPi in healthy athymic nude mice (Additional file 1: Figure S6). The tracer was quickly cleared from the blood, similar to other inhibitors of this type [2, 26, 27], with an alpha blood half-life of 14.3 min (96.25 %) and beta blood half-life of 94.6 min (3.75 %), resulting in a weighted blood half-life of *t*_1/2_(weighted) = 17.1 min (Additional file 1: Figure S6). The biodistribution of [^131^I]-I2-PARPi was analyzed after administration of the radiolabeled tracer (24 ± 5 μCi) in U87 MG tumor-bearing mice (Fig. 7a, b). Administration of [^131^I]-I2-PARPi with a specific activity of 50 mCi/μmol showed the best tumor/muscle and tumor/brain ratios (Fig. 7a, Additional file 1: Table S1), which is why this specific activity was selected for further in vivo imaging experiments. Administration of [^131^I]-I2-PARPi with a specific activity of 50 mCi/μmol showed the best tumor/muscle and tumor/brain ratios (Fig. 7a, Additional file 1: Table S1). Figure 7b compares the biodistribution of the radiotracer after different time points. The most favorable tumor/muscle and tumor/brain ratios were seen at 2 h post intravenous injection (0.50 ± 0.08 %ID/g, 0.04 ± 0.02 %ID/g and 0.007 ± 0.002 %ID/g for tumor, muscle, and brain, respectively). Figure 7 shows selected tissues, and a full biodistribution table is shown in Additional file 1: Table S2.

### In vivo imaging and autoradiography

**Fig. 8:**
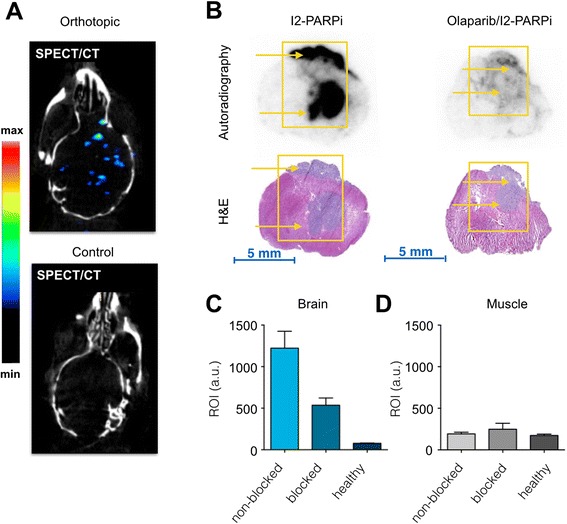
Uptake of [^131^I]-I2-PARPi in orthotopic U251 MG brain tumors. **a** SPECT/CT images of a mouse bearing an orthotopic U251 MG brain tumor (*top*) and a healthy mouse treated with [^131^I]-I2-PARPi (*bottom*). **b** Autoradiography and H&E stains of ex vivo brain sections from orthotopic U251 MG brain tumor mice. Mice were treated with [^131^I]-I2-PARPi alone (*left*) or [^131^I]-I2-PARPi with a pre-injection of Olaparib (*right*). *Yellow arrows* point towards the tumors. **c**, **d** Quantification of [^131^I]-I2-PARPi uptake in brain or muscle in non-blocked, blocked, and healthy mice

With the aim of determining the potential of radioiodinated I2-PARPi as a glioblastoma imaging agent, small animal SPECT/CT and PET/CT studies were performed in athymic nude mice bearing orthotopic U251 MG xenografts. SPECT/CT studies were performed using [^131^I]-I2-PARPi, and PET/CT studies were performed with the structural analog [^124^I]-I2-PARPi. In SPECT/CT, acquired 90 min post injection of the [^131^I]-I2-PARPi, the orthotopic tumor could be readily visualized, with uptake of the tracer in the right hemisphere of the brain, where the tumor was implanted (Fig. 8a). This data is also supported by ex vivo autoradiography. Histological sections of orthotopic U251 MG tumors showed a clear delineation of tumor tissue with [^131^I]-I2-PARPi, but not for mice where PARP1 was saturated with a pre-injection of the non-labeled PARP1 inhibitor Olaparib (Fig. 8b). The signal intensity of [^131^I]-I2-PARPi in tumor tissue of mice that received no Olaparib was 15.7-fold higher than healthy brain tissue (Fig. 8c) and 6.2-fold higher than muscle tissue (Fig. 8d), corroborating the SPECT data. There was a 65 % reduction in the intensity of the autoradiography signal in tumor tissue for mice that had been treated with Olaparib (1222 ± 203 and 536 ± 87 AU for mice without and with Olaparib treatment, respectively), further verifying the specificity of the compound. In contrast, the intensity of the muscle did not undergo statistically significant changes (192 ± 20 and 248 ± 72 AU for mice without and with Olaparib treatment, respectively, Fig. 8d).

**Fig. 9:**
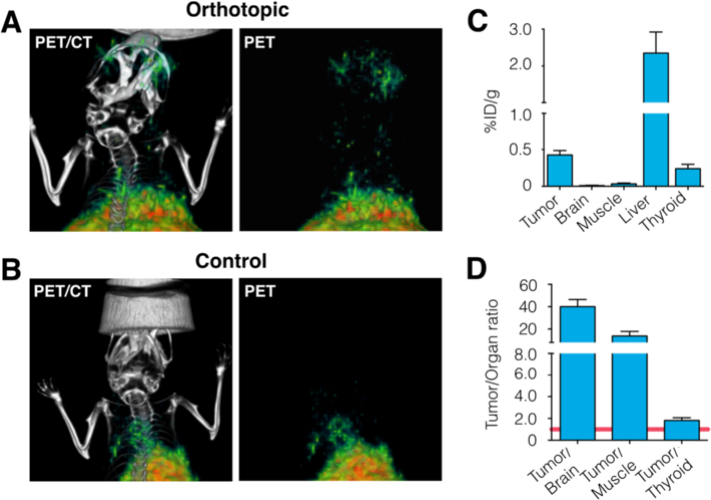
PET imaging of orthotopic brain tumors with [^124^I]-I2-PARPi. **a** PET/CT coronal images (le*ft*) and corresponding PET images (*right*) of orthotopic U251 MG brain tumor mice injected with [^124^I]-I2-PARPi. **b** PET/CT coronal images (*left*) and corresponding PET images (*right*) of a healthy mouse treated with [^124^I]-I2-PARPi. **c** Biodistribution of [^131^I]-I2-PARPi in a U251 MG xenograft mouse model (mice were sacrificed 2 h after tracer injection, additional biodistribution data can be found in Additional file 1: Tables S3A and S3B). **d** Selected tumor to non-target tissues ratios of [^131^I]-I2-PARPi. Radioactivity in tissues is expressed as %ID/g

PET/CT data was obtained after intravenous injection 180–230 μCi of [^124^I]-I2-PARPi (110–170 mCi/μmol). Similar to SPECT/CT, orthotopic U251 MG xenografts were clearly visualized non-invasively, whereas healthy mice showed negligible uptake of the tracer (Fig. 9a). Ex vivo biodistribution data with [^131^I]-I2-PARPi corroborated the PET/CT data (Fig. 9b and Additional file 1: Table. S3A). Comparably to U87 MG, we determined the tumor uptake in U251 MG to be 0.43 ± 0.05 %ID/g, whereas only a minute amount of tracer was retained in the healthy brain (0.011 ± 0.003 %ID/g). High uptake was observed in the liver (2.4 ± 0.6 %ID/g), which is common for intravenously administered small molecules that are excreted hepatobiliary. The tumor/brain ratio was found to be 40.0 ± 6.3, and the tumor/muscle ratio was 13.7 ± 4.1 (Fig. 9c and Additional file 1: Table S3B) indicating potential clinical value of the tracer.

## Discussion and conclusions

Glioblastoma multiforme (GBM) is characterized by aggressive malignant infiltrative growth and is associated with a dismal prognosis [28]. Current standard of care for non-invasive glioblastoma diagnosis is MRI [29, 30], which often offers acceptable information regarding the size and shape of the tumor. However, this tool is often unable to characterize the underlying histopathology of the disease. Better and more accurate tools are therefore desperately needed, particularly for detecting glioblastoma at low levels of infiltration [8]. The first step of this work was the development of a library of novel, iodinated small molecules, which are targeted to PARP1 via their 2H-phthalazin-1-one group. Biochemical assays and basic pharmacokinetic evaluation showed that some of the small molecules had IC_50_ values close to 10 nM (9 ± 2 nM and 11 ± 3 nM for I2-PARPi and I1-PARPi), which is close to the original Olaparib [22] and lower than other well-performing in vivo PARP1 imaging agents [26, 27]. The small molecule I2-PARPi, derived from 4-iodobenzoic acid, demonstrated the best in vitro PARP1 binding and the optimal biophysical properties for further use in in vivo (IC_50_ = 9 ± 2 nM, CHI = 59.6, log*P*_CHI_, plasma free fraction = 11.5 ± 0.1 %; Fig. 3b). These pharmacokinetic properties were complemented in vitro and in vivo by competition studies of the non-radiolabeled I2-PARPi and the fluorescent PARPi-FL [26, 27]. Fluorescence-based in vitro assays confirmed selective nuclear accumulation of all our compounds in U87 MG glioblastoma cells (Fig. 5), where uptake of a fluorescent PARP1 inhibitor was inhibited in the presence of the iodo-PARP1 agents, leading to a significant reduction in nuclear fluorescence intensity between 76 ± 6 and 67 ± 13 %. This reduction was comparable to the parent scaffold Olaparib (73 ± 11 %). Similarly, in vivo injection of I2-PARPi reduced the uptake of the fluorescent probe equally well, confirming the ability of the iodinated small molecule to bind to PARP1, and to target the enzyme inside of cell nuclei. The radiolabeled analog of this inhibitor was obtained through nucleophilic substitution of the (Bu_3_)Sn labeled cold precursor with ^131^I or ^124^I in mild conditions with a specific activity between 145–210 and 110–170 mCi/μmol for [^131^I]-I2-PARPi and [^124^I]-I2-PARPi, respectively (Fig. 4a). The tracer showed a weighted blood half-life *t*_1/2_ of 17.1 min (Additional file 1: Figure S6), a typical pharmacokinetic profile for small molecules, which matches up with values obtained for other Olaparib derivatives [2, 23]. In vivo biodistribution studies of [^131^I]-I2-PARPi in U251 MG subcutaneous xenograft models (Additional file 1: Table S3) confirmed rapid clearance from all organs via the liver (2.3 ± 0.6 %ID/g; Fig. 9c). A significant accumulation of the tracer in glioblastoma tissue (0.43 ± 0.05 %ID/g; Fig. 9c) compared to other control tissues like brain (0.011 ± 0.003 %ID/g) or muscle (0.033 ± 0.012 %ID/g) was observed, leading to a signal/noise ratio of 40.05 ± 6.34 (Fig. 9d), ideal for in vivo imaging. The difference in tumor to brain uptake was also verified by autoradiography, where the [^131^I]-I2-PARPi uptake was 15.7-fold higher than that in the healthy brain tissue (Fig. 8b). At the same time, the PARP1 specificity of our tracer was confirmed after treatment with the PARP1 inhibitor Olaparib (Fig. 8c) showing a significant drop in signal intensity. The potential value of I2-PARPi as a tracer for clinical imaging of glioblastoma was corroborated in an orthotopic U251 MG glioblastoma mouse model by both SPECT/CT and PET/CT imaging (Figs. 8 and 9), where a clear accumulation of the tracer in the tumor tissue could be observed.

In summary, we have designed, radiolabeled, and tested a library of iodinated PARP1 inhibitors, based on the high affinity of PARP1 to the 2H-phthalazin-1-one scaffold. The high tolerability of PARP1 for small molecules appended to 2H-phthalazin-1-one resulted in a number of high affinity binders in our small molecule library, the best representative of which, [^124/131^I]-I2-PARPi, was tested as a PET and SPECT tracer. Our data demonstrates the specific binding of our I2-PARPi tracer to PARP1 and illustrates the potential of this tracer for glioblastoma detection.

## Additional file

Additional file 1:
**Supporting information.** This file contains supplemental figures S1 to S6 and supplementary tables S1 to S3. (DOCX 661 kb)

## Abbreviations

AcN: acetonitrile
CHI: chemical hydrophobicity index
EDC: 1-ethyl-3-(3-dimethylaminopropyl) carbodiimide
HBTU: *N*,*N*,*N*′,*N*′-tetramethyl-*O*-(1H-benzotriazol-1-yl) uronium hexafluorophosphate
NHS: *N*-hydroxysuccinimide
