# Supplementary material for: Radioiodinated PARP1 tracers for glioblastoma imaging
Source: EJNMMI Res. 2015 Sep 4;5:46. doi: 10.1186/s13550-015-0123-1 (PMC4559561; doi:10.1186/s13550-015-0123-1)
Supplement: Additional file 1: — Supporting information. This file contains supplemental figures S1 to S6 and supplementary tables S1 to S3. (DOCX 661 kb) [file 13550_2015_123_MOESM1_ESM.docx]

**- Supporting Information -**

**Radioiodinated PARP1 Tracers for Glioblastoma Imaging**

Beatriz Salinas, ^1^ Christopher P Irwin,^1^ Susanne Kossatz,^1^ Alexander Bolaender,^2^ Gabriela Chiosis,^2^ Nagavarakishore Pillarsetty,^1^ Wolfgang A Weber,^1,2,3^ Thomas Reiner^1,3,*^

^1^ Department of Radiology and ^2^ Program in Molecular Pharmacology, Memorial Sloan Kettering Cancer Center, New York, New York 10065, USA

^3^ Weill Cornell Medical College, New York, NY, 10065, USA

Correspondence:

* Thomas Reiner, Ph.D.

Department of Radiology
Memorial Sloan Kettering Cancer Center
1275 York Avenue
New York, NY 10065

USA
Ph. 646 888 3461

reinert@mskcc.org reinert@mskcc.org

Table of Contents

Supplementary figure S13

Supplementary figure S24

Supplementary figure S35

Supplementary figure S46

Supplementary figure S57

Supplementary figure S68

Supplementary table S19

Supplementary table S210

Supplementary table S311

**
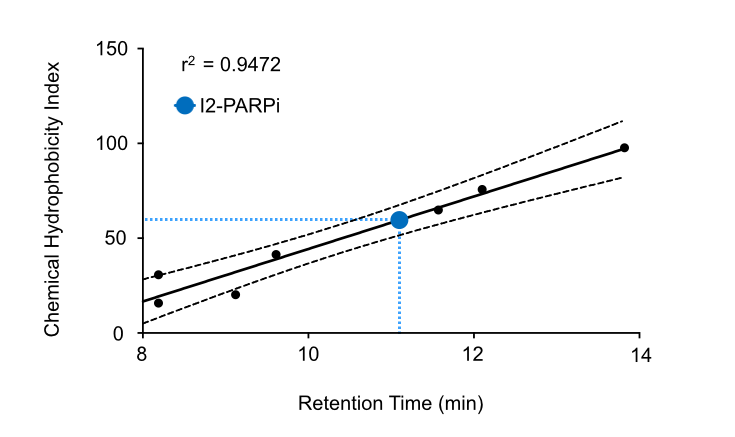
**

**Figure S1.** Chemical Hydrophobicity Index (CHI) calibration curve obtained from HPLC retention times of standards with known CHI values. Blue datapoint: CHI value calculated for I2-PARPi based on the HPLC retention time (RT = 11.1 min, CHI = 59.6. Compounds used for the calibration were: theophylline (RT = 8.2 min), 5-phenyl-1H-tetrazole (RT = 9.1 min), benzimidazole (RT = 8.2 min), colchicine (RT = 9.6 min), acetophenone (RT = 11.6 min), indole (RT = 12.1 min), valerophenone (RT = 13.8 min).

**
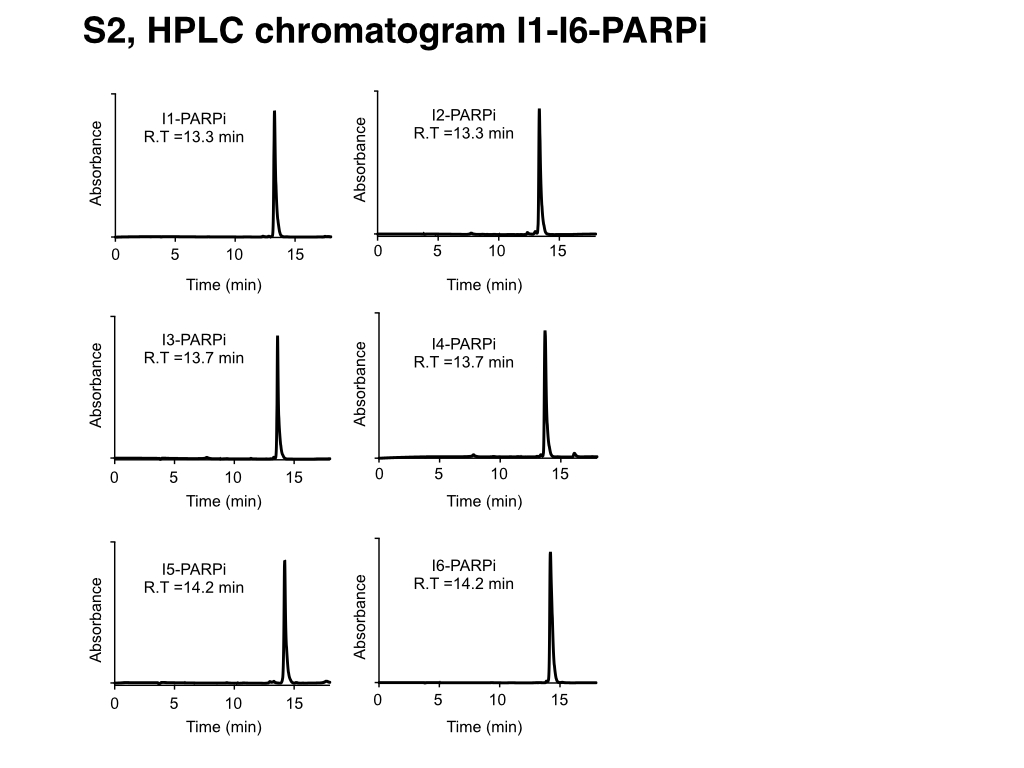
**

**Figure S2.** HPLC chromatograms of purified Iodo-PARPi inhibitors (254 nm) on a reversed phase Atlantis T3 column (C18, 5 μm, 4.6 mm × 250 mm, flowrate: 1.0 mL/min, Solvents: Water (A) and Acetonitrile (B). Gradient: 5% - 95% B (0 min -15 min); 95% B (15 min -17 min); 95% - 5% B (17 min - 18 min).

**
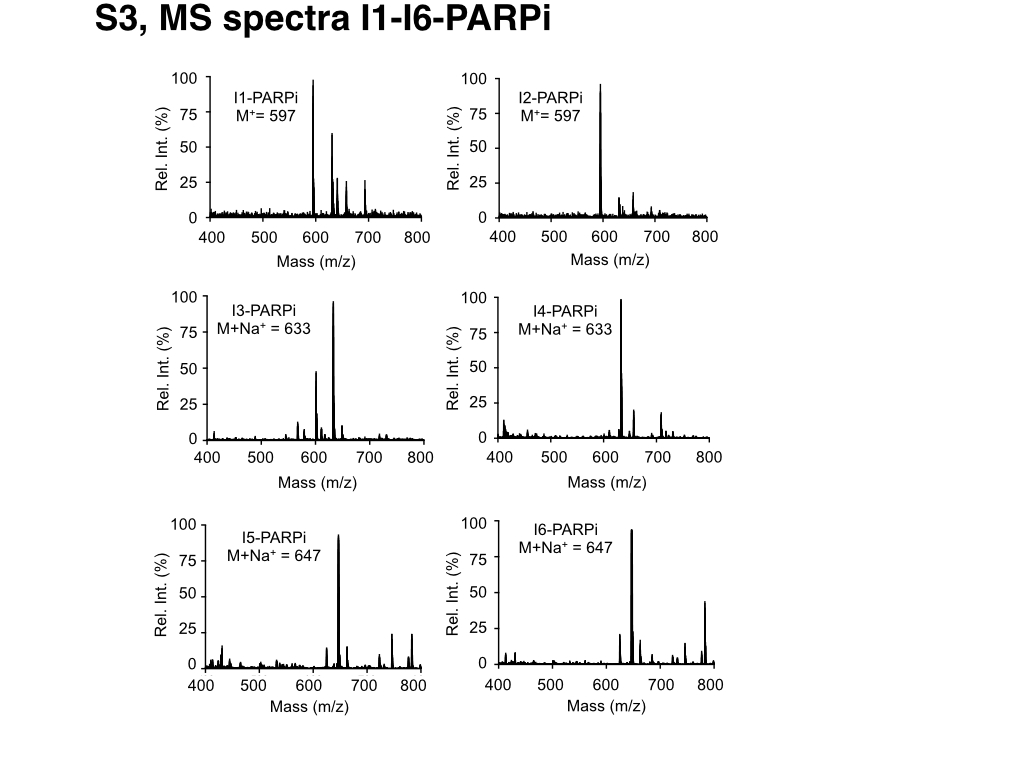
**

**Figure S3.** LC-ESI-MS spectra of purified Iodo-PARPi inhibitors.

**
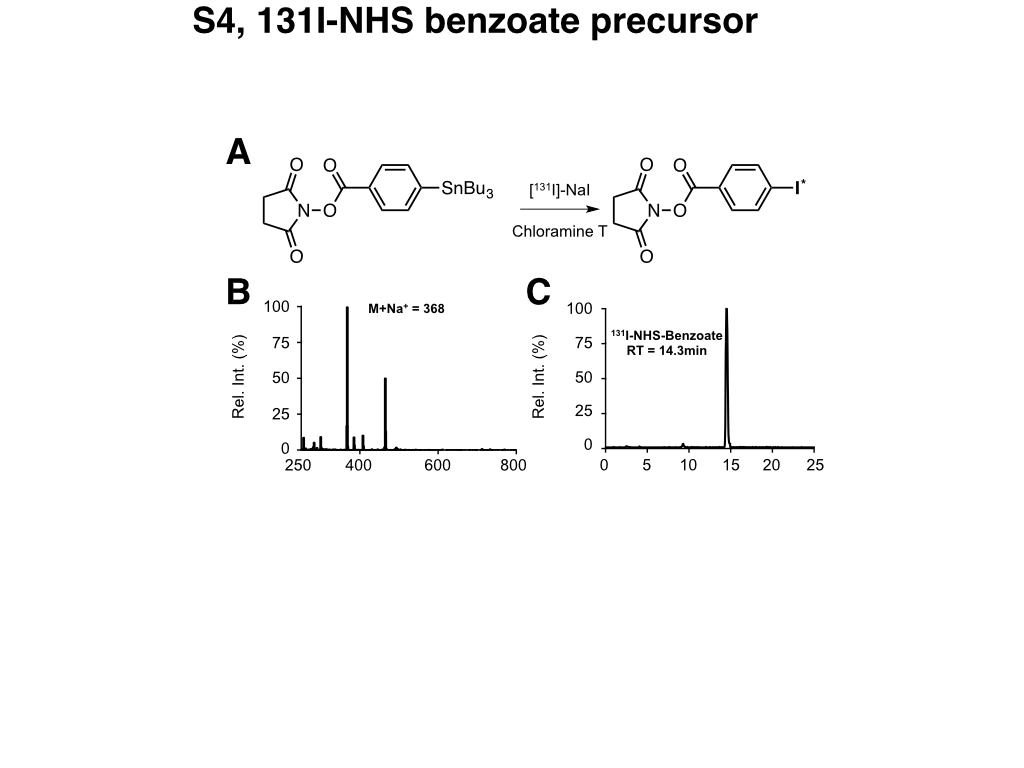
**

**Figure S4.** Radiochemical synthesis of the precursor [^131^I]-NHS-benzoate. (A) Coupling reaction with SnBu_3_-NHS-benzoate and [^131^I]-NaI; (B) Mass spectrometry spectra of [^131^I]-NHS-benzoate; (C) HPLC chromatogram of [^131^I]-NHS-benzoate radio-labeled precursor (radiotrace).

**
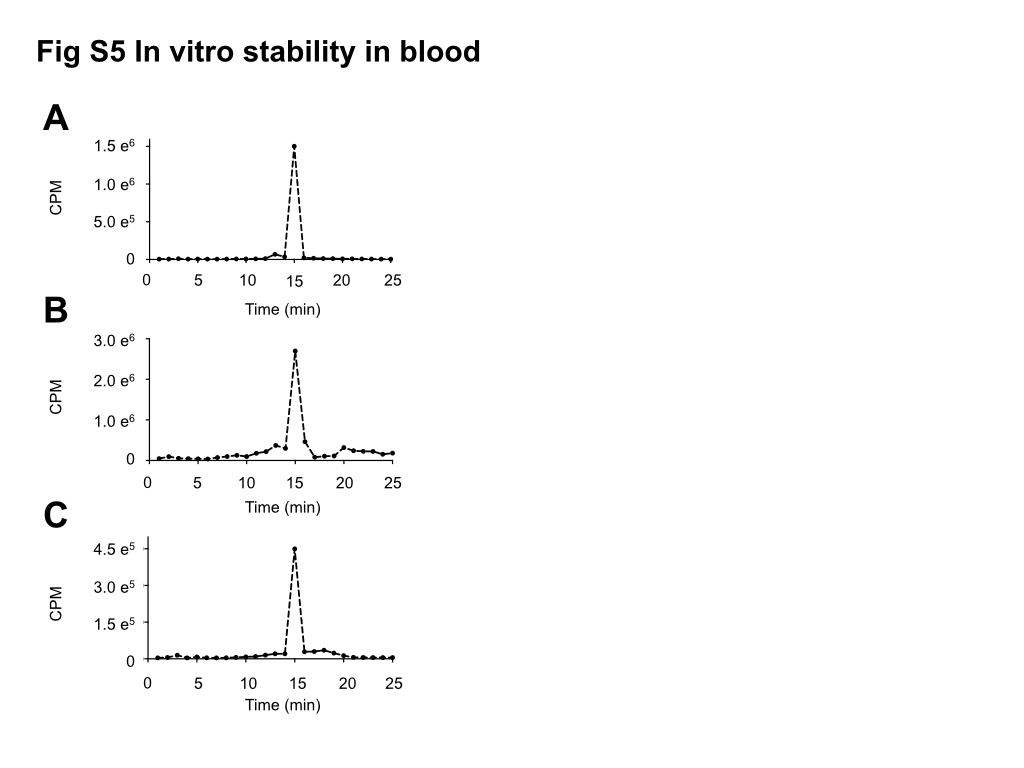
**

**Figure S5.** In vitro stability of [^131^I]-I2-PARPi incubated in mouse blood for 0 min (A), 60 min (B), and 120 min (C) at 37 °C. Supernatants were analyzed via HPLC and fractions collected every minute. Radioactivity of the fractions was counted using a gamma counter


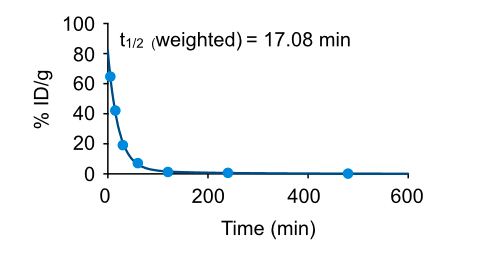


**Figure S3.** *Ex vivo* blood half-life of ^131^I-PARPi (n=3). Mice were injected with ^131^I-PARPi (50 μCi in 200 μL PBS/PEG_300_ (10:1)) and blood samples collected at different time points (5, 15, 30, 60, 120, 240, and 480, min), weighed and activity determined using a gamma counter. Results expressed as %injected dose/gram (%ID/g).


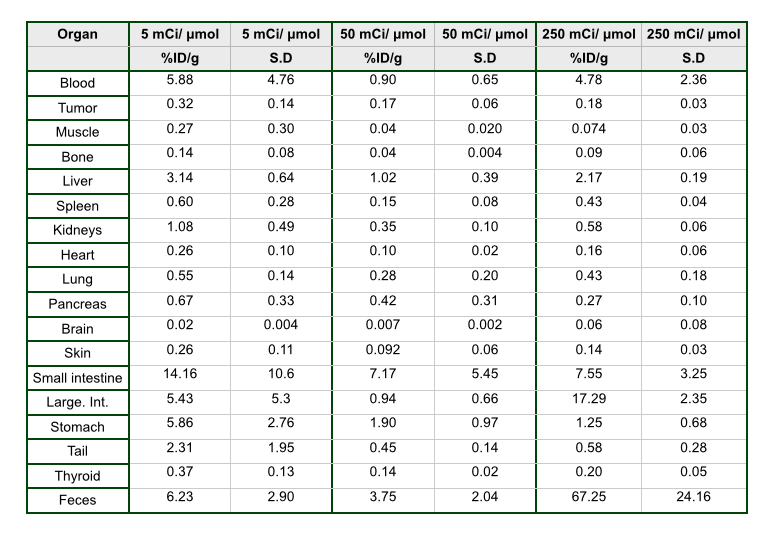


**Table S1.** Biodistribution of ^131^I-I2-PARPi in U87 MG xenograft mouse models. Mice were sacrificed at 2h post injection of 20-30 μCi of ^131^I-I2-PARPi in 200 μL of a solution 90% PBS 10% PEG_300_ with different specific activities (5, 50 and 250 mCi/ μmol). Values are plotted as %ID/g. SD represents standard deviation. Select organs are shown in Fig. 7A.


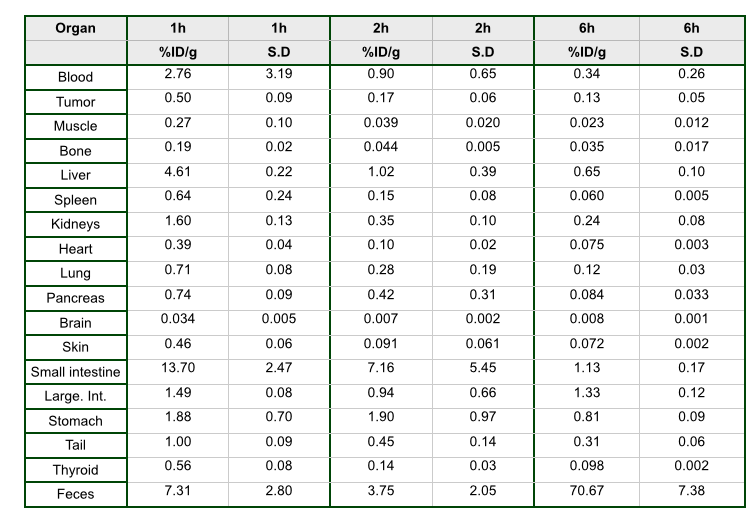


**Table S2.** Biodistribution of ^131^I-I2-PARPi in U87 MG xenograft mouse models. Mice were sacrificed at different time points (1h, 2h and 6h) post injection of 20-30 μCi of ^131^I-I2-PARPi in 200 μL of a solution 90% PBS 10% PEG_300_. Values are plotted as %ID/g. SD represents standard deviation. Select organs are shown in Fig 7B.


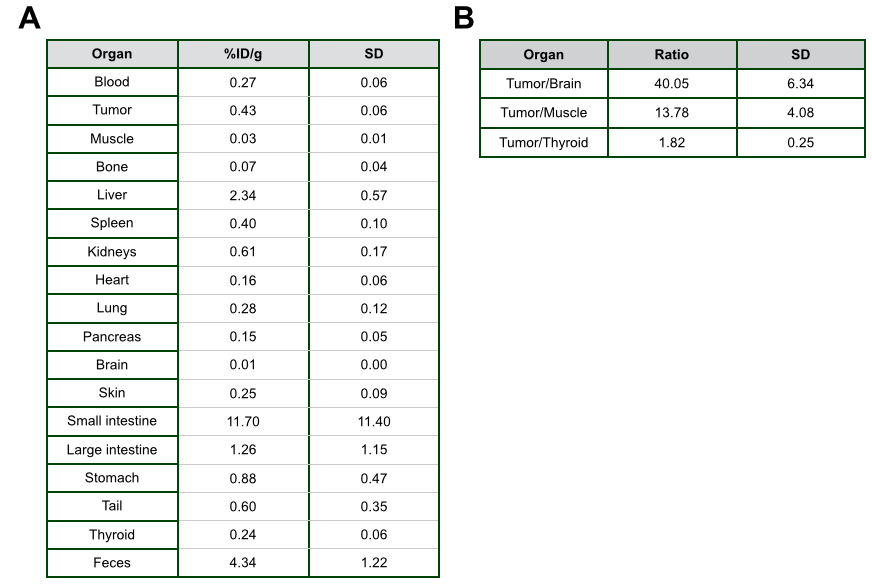


**Table S3.** Biodistribution of ^131^I-I2-PARPi in U251 MG xenograft mouse models. A) Mice were sacrificed at 2h post injection of 20-30 μCi of ^131^I-I2-PARPi in 200 μL of a solution 90% PBS 10% PEG_300._ Select organs are shown in Fig. 9C-B) Selected tumor to non-target tissues ratio for ^131^I-I2-PARPi.
